# Supplementary material for: Preferential Expression of Programmed Death Ligand 1 Protein in Tumor-Associated Macrophages and Its Potential Role in Immunotherapy for Hepatocellular Carcinoma
Source: Int J Mol Sci. 2021 Apr 29;22(9):4710. doi: 10.3390/ijms22094710 (PMC8124544; doi:10.3390/ijms22094710)
Supplement: Supplementary file 1 [file ijms-22-04710-s001.zip › ijms-1177565-supplementary.pdf]

Supplementary Figure 1

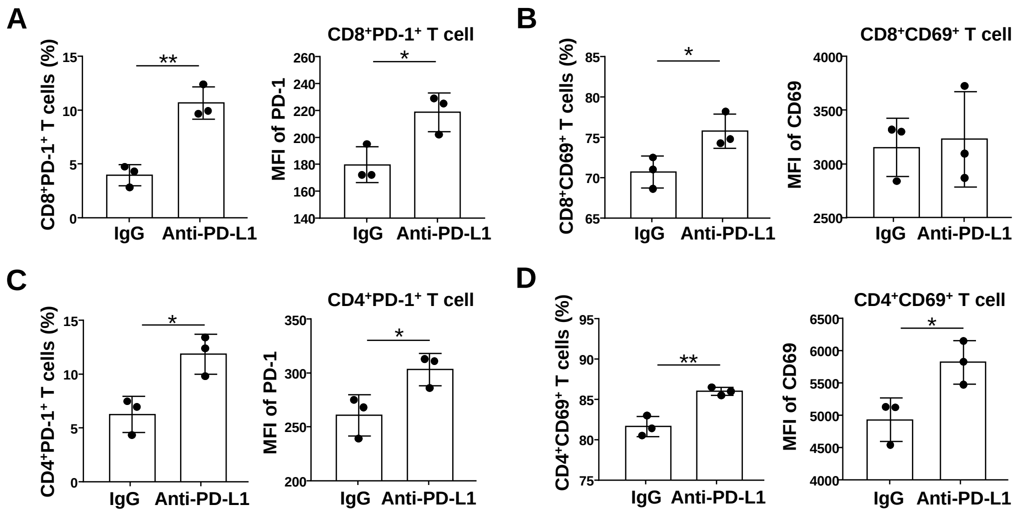

Supplementary Figure 1. PD-1, CD69 expression on PMA/Ionomycin-activated CD8$^+$ and CD4$^+$ T cells increased significantly after PD-L1 blocking in M2 macrophages

A. CD8$^+$ PD1$^+$ T cells frequency and MFI
B. CD8$^+$ CD69$^+$ T cells frequency and MFI
C. CD4$^+$ PD1$^+$ T cells frequency and MFI
D. CD4$^+$ CD69$^+$ T cells frequency and MFI

Supplementary Figure 2

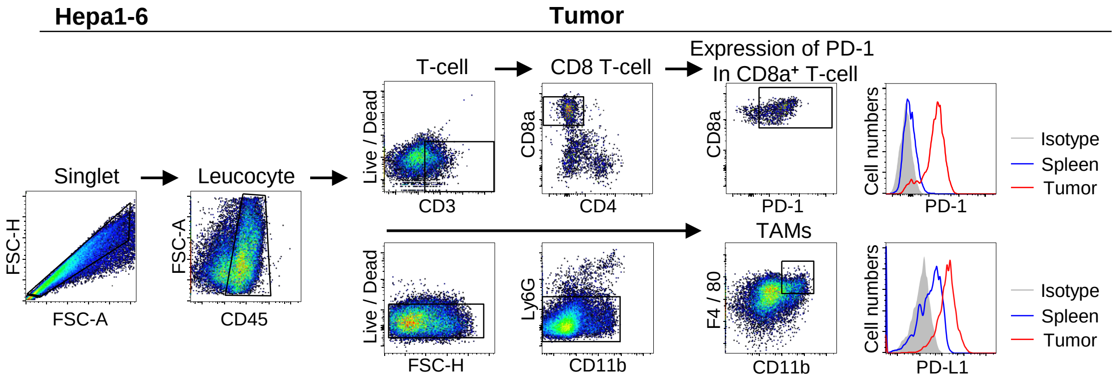

Supplementary Figure 2. Flow cytometry gating strategy in a hepa1-6 syngeneic mouse model.

Supplementary Figure 3

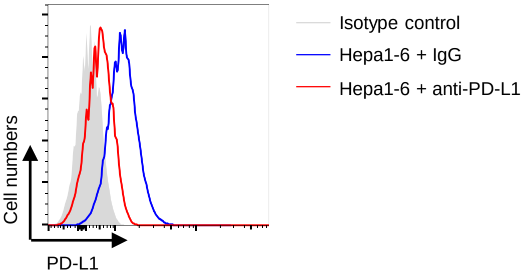

Supplementary Figure 3. PD-L1 expression in Hepa1-6 cells.

Supplementary Table 1
Antibodies and protein used in this study

Antibodies	Clone	Company	Dilution	Target host	Application
**Anti-CD3**		Abcam	1:100	Human	IHC
**Anti-CD68**	KP1	Abcam	1:1000	Human	IHC
**Anti-PD-L1**	22C3	Dako	2μg/ml	Human	IHC
**Anti-PD-L1 antibody**	29E.2A3	BioXcell	20μg/ml	Human	Blocking
**Anti-PD-L1 antibody**	10F.9G2	BioXcell	100μg/ml	Mouse	Blocking
**Normal mouse IgG**		Santacruz	100μg/ml	Human	Blocking control
**Normal rat IgG2b**		Santacruz	100μg/ml	Mouse	Blocking control
**Live/Dead Fixable aqua Dead Cell Stain Kit**		Invitrogen	1μl/10^6 cells	Human, Mouse	Flow cytometry
**PerCP-Cy5.5-conjugated anti-CD3**	UCHT1	BD	1μl/10^6 cells	Human	Flow cytometry
**V450-conjugated anti-CD8**	RPA-T8	BD	1μl/10^6 cells	Human	Flow cytometry
**APC-H7-conjugated anti-CD4**	SK3	BD	1μl/10^6 cells	Human	Flow cytometry
**PE-Cy7-conjugated anti-Ki-67**	B56	BD	1μl/10^6 cells	Human	Flow cytometry
**APC-conjugated anti-CD69**	FN50	BD	1μl/10^6 cells	Human	Flow cytometry
**PE-conjugated anti-PD-1**	NAT105	Biolegend	1μl/10^6 cells	Human	Flow cytometry
**PE-Cy7-conjugated anti-Ly6G**	1A8	BD	1μl/10^6 cells	Mouse	Flow cytometry
**APC-conjugated anti-PD-L1**	MIH1	BD	1μl/10^6 cells	Human	Flow cytometry
**V500-conjugated anti-CD45**	30-F11	BD	1μl/10^6 cells	Mouse	Flow cytometry
**APC-conjugated anti-CD69**	H1.2F3	BD	1μl/10^6 cells	Human	Flow cytometry
**V450-conjugated anti-CD11b**	M1/70	BD	1μl/10^6 cells	Mouse	Flow cytometry
**BV421-conjugated anti-CD3**	17A2	BD	1μl/10^6 cells	Mouse	Flow cytometry
**APC-Cy7-conjugated anti-F4/80**	BM8	Biolegend	1μl/10^6 cells	Mouse	Flow cytometry
**V500-conjugated anti-CD3**	UCHT1	BD	1μl/10^6 cells	Human	Flow cytometry
**PerCP-Cy5.5-conjugated anti-CD8a**	53.6.7	ebioscience	1μl/10^6 cells	Mouse	Flow cytometry
**PE-conjugated anti-IFN-γ**	B27	BD	1μl/10^6 cells	Human	Flow cytometry
**PE-Cy7-conjugated anti-TNFα**	MAb11	BD	1μl/10^6 cells	Human	Flow cytometry
**Recombinant human IL-4**		R&D systems	1μg/ml	Human	M2-polarization
**Recombinant human M-CSF**		R&D systems	1μg/ml	Human	M2-polarization
